# Optimization of the optimal hormone formula for kiwifruit and analysis of its storage characteristics

**DOI:** 10.3389/fpls.2025.1567183

**Published:** 2025-05-09

**Authors:** Xiaoe He, Wei Qin, Qi He, Yunxiang Liu, Yan Wang, Rencai Wang, Hao Shi

**Affiliations:** ^1^ College of Agriculture and Forestry Science and Technology, Hunan University of Applied Technology, Changde, Hunan, China; ^2^ Institute of Economics and Business, Hunan Biological Electromechanical Vocational Technical College, Changsha, Hunan, China; ^3^ College of Horticulture, Hunan Agricultural University, Changsha, Hunan, China; ^4^ College of Life and Environmental Sciences, Hunan University of Arts and Sciences, Changde, Hunan, China

**Keywords:** kiwifruit, hormones, hardness, quality, orthogonal experiment, metabolomic, transcriptomic

## Abstract

**Introduction:**

Kiwifruit is susceptible to ripening and senescence during postharvest storage, leading to fruit softening, rotting, and nutrient loss, affecting commercial and economic values. Hormones delay senescence by regulating fruit physiology and metabolism, but their specific effects and mechanisms must be further investigated.

**Methods:**

To extend the postharvest storage duration of kiwifruit, we conducted a study using 'Yan Nong 3' kiwifruit as our test material. The fruits were treated with varying concentrations of Brassinolide (BRs), melatonin (MT), methyl jasmonate (MeJA) and salicylic acid (SA), respectively. Subsequently, on the basis of a one-way test, an orthogonal experiment was designed with fruit hardness as an indicator (7 days of storage at room temperature) to obtain the optimal process formulation for phytohormone synergistic treatment (PEHC): 10 μmol·L^-1^ for BRs, 200 μmol·L^-1^ for MT, 300 μmol·L^-1^ for MeJA, and 2 mmol·L^-1^ for SA.

**Results:**

The results showed that after 60 days of storage at 4°C, PEHC was found to increase the good fruit rate and the hardness of kiwifruit by 5.97% and 67.42%, respectively, compared to the control. PEHC reduced weight loss rate and disease index, slowed the decrease in titratable acid content (TAC) and vitamin C (VC) content, maintained color, and delayed the accumulation of SSC. At 80 days of storage, the hardness, good fruit rate, VC content, and TAC of PEHC increased by 68.38%, 28.87%, 32.76 mg·100 g^-1^, and 20.00%, respectively, compared to the control. Whereas, the difference in SSC of PEHC compared to control was nosignificant. The PEHC reduced the content of 1-Aminocyclopropane-1-carboxylate (ACC). Transcriptomics revealed that PEHC inhibited the gene expression levels of *Acc08469* in s-adenosylmethionine synthetase (MetK), as well as *Acc20538, Acc24995*, and *Acc17490* in 1-aminocyclopropane-1-carboxylic acid oxidase. Using metabolomics, PEHC increased the relative contents of acids and amino acids and decreased the relative contents of aroma, pigments or phenolics, and soluble sugars compared with the control, of which the trends of changes in acids and soluble sugars were consistently associated with the changes in fruit quality.

**Discussion:**

The PEHC had a favorable effect on maintaining kiwifruit quality and delayed the decline in postharvest storage quality.

## 1 Introduction

Kiwifruit is a deciduous vine. In 2022, the total kiwifruit production in China was 2.38 million tons, accounting for 52.4% of the global total and ranking first in the world (Zhang et al., 2024). Kiwifruit is popular among the public and has high nutritional and economic value due to its high content of minerals, vitamins C and E, dietary fiber, and amino acids (Satpal et al., 2021; Wang et al., 2021). In recent years, the kiwifruit industry in China has been developing rapidly and has become a pillar industry for agricultural development and rural revitalization, playing an important role in promoting farmers’ income, accelerating the structural adjustment of the fruit industry, and alleviating poverty. Kiwifruit is a typical climacteric and ethylene-sensitive fruit; it is prone to softening, dehydration-induced shrinkage, and quality loss under room-temperature storage, eventually leading to fruit rot and spoilage (Gwanpua et al., 2018; Wang et al., 2020). Kiwifruit faces problems, including a more concentrated time to market, bad fruit in storage, and a short shelf-life after leaving the warehouse, making it particularly important to improve post-harvest fruit storage technology.

Currently, the primary preservation techniques employed for kiwifruit include low-temperature refrigeration, air-conditioned physical storage, packaging storage, and pharmaceutical preservation methods (such as vitamin C, Chitosan, and 1-methylcyclopopene, etc). Notably, research on plant hormone preservation has emerged as a popular research topic in recent years, due to its pollution-free nature and the difficulty in developing resistance to it. Among them, the preservation effect of synergistic treatment with compound hormones was better than that after single hormone treatment. Yin et al. (2013) demonstrated that SA and its derivative, acetylsalicylic acid, are crucial plant hormones capable of regulating the expression of genes related to ethylene synthesis and signaling. By inhibiting ethylene synthesis, these hormones can effectively delay the ripening process of the fruit. Hu M and Luo et al. (Hu, 2018; Lou et al., 2022) discovered that the application of exogenous melatonin (MT) during kiwifruit storage can effectively slow down the increase in fruit hardness, TAC, and SSC, while also inhibiting the decline in respiration rate. This, in turn, maintains the fruit’s storage quality and significantly extends its shelf life. Lai and Pan et al. (Li et al., 2019; Pan et al., 2020) further demonstrated that the use of MeJA can effectively inhibit the postharvest decline in kiwifruit hardness, VC content, SSC, and soluble sugar content. Additionally, it exhibits a notable preventive and curative effect in reducing the incidence of fruit diseases.

Niu et al. (2023) found that MeJA combined with SA treatment effectively inhibited the reduction of fruit firmness, delayed the increase of decay rate and disease index, and reduced the accumulation of soluble solids compared with single hormone treatment. Yu et al. (Yu and Xie, 2019) concluded that combining multiple preservation methods not only outperformed pure preservation techniques in terms of effectiveness but also complemented each other’s deficiencies, thereby maximizing the retention of nutrients within the fruit. Despite this, research on the synergistic effects of the hormones BRs, MT, MeJA, and SA on the storage quality of postharvest kiwifruit remains scarce. Transcriptomics is a discipline that studies the transcription and transcriptional regulation of genes in cells at the global level. Metabolomics is a discipline that studies the collection of all metabolites within a cell at a given moment. In this study, ‘Yan Nong 3’ kiwifruit was selected as the test material. The fruit is elliptical, with brown skin and short fuzz. The flesh is dark green. Through a series of one-way and orthogonal tests, an optimized hormone compound formulation was developed. Conduct relevant quality research on this optimized hormone compound formulation, and combine transcriptomics and metabolomics analysis to investigate the differences in metabolite composition and content in kiwifruit, as well as the expression levels of related genes. The impact of these optimal hormone combinations on the quality of post-harvest kiwifruit was then investigated, aiming to provide a scientific basis for the regulation of postharvest storage quality in kiwifruit.

## 2 Materials and methods

### 2.1 Test material

Kiwifruits of the “Yan Nong 3” variety were harvested from Hunan Tao Yuan Ming Fruit Biological Company, located in Taoyuan County, Hunan Province. After harvest, kiwifruits were transported back to the laboratory on the same day to ensure freshness and preserve their quality for further analysis.

### 2.2 Test method

#### 2.2.1 Optimization of the hormone compound formula for kiwifruit

(1) One-way test

On September 17, 2023, kiwifruit were picked in the orchard and transported back to the laboratory. Kiwifruits, devoid of mechanical damage and disease spots, and exhibiting uniformity in both size and maturity (with an initial mean hardness of 8.84 kg·cm^-2^ and a mean SSC of 6.44%), were meticulously selected. The effects of various concentrations of brassinolide (1, 5, 10, 15, 20 μmol·L^-1^), melatonin (50, 100, 200, 300, 500 μmol·L^-1^), methyl jasmonate (50, 100, 200, 300, 400 μmol·L^-1^), and salicylic acid (0.5, 1, 2, 4, 6 mmol·L^-1^) on postharvest weight loss rate, good fruit rate, hardness, and SSC of kiwifruit were investigated (**Table 1**). The experiment included a control treated with distilled water. The kiwifruit was soaked for 2 minutes according to the different concentration treatments, then dried until the outer skin was free of moisture, and subsequently stored at room temperature for 7 days to determine the indexes.

**Table 1:** Kiwifruit hormone treatment levels.

Level	Factor			
	Brassinolide (μmol·L^-1^) A	Melatonin (μmol·L^-1^) B	Methyl jasmonate (μmol·L^-1^) C	Salicylic acid (mmol·L^-1^) D
1	1	50	50	0.5
2	5	100	100	1
3	10	200	200	2
4	15	300	300	4
5	20	500	400	6

(2) Orthogonal test

Drawing from the results of the one-way test, three successive optimal concentrations were chosen from among the five levels of each hormone for the Lg(3^4^) orthogonal experimental design (**Table 2**). The hardness of the kiwifruit served as the key investigation index to ascertain the most effective hormone formulation.

**Table 2:** Orthogonal experimental design table.

Factor				
Level	Brassinolide (μmol·L^-1^) A	Melatonin (μmol·L^-1^) B	Methyl jasmonate (μmol·L^-1^) C	Salicylic acid (mmol·L^-1^) D
-1	5	200	100	0.5
0	10	300	200	1
1	15	500	300	2

#### 2.2.2 Storage validation test of kiwifruit fruits treated with optimal hormone formulation

On October 2, 2023, kiwifruit were picked in the orchard and transported back to the laboratory. Kiwifruits, devoid of mechanical damage and disease spots, and exhibiting uniformity in both size and maturity (with an initial mean hardness of 8.62 kg·cm^-2^ and a mean SSC of 6.87%), were meticulously selected. There, they were soaked for 2 mins using the optimal hormone formulation, then dried until the outer skin was free of moisture. The kiwifruit were stored at room temperature for 7 days to determine their hardness.

#### 2.2.3 Experiment on the effect of optimum hormone formulation treatment on the storage quality of kiwifruit fruits

Kiwifruit fruits of similar hardness, size, fruit shape, and maturity were picked on October 14, 2023, on a clear morning, in batches of pre-cooled plastic baskets, and immediately brought back to the laboratory. The initial average hardness of kiwifruit harvested on the same day was 8.51 kg·cm^-2^, with an average SSC of 8.13%. The test was conducted by soaking kiwifruit in the solution made from the optimal hormone formulation for 2 mins, then they were drained, divided into preservation bags (made of polyethylene), and stored at 4°C. A control group (Control) was set up with distilled water as the treatment. There were two treatments in total. Each treatment group consisted of 150 kiwifruit, divided into three batches of 50 each. Fruit quality was measured starting on the day of storage and every 10 days thereafter. In this paper, the optimal hormone formulation treatment is referred to as plant endogenous hormone complex (PEHC) hereafter.

#### 2.2.4 Indicator measurement

(1) Weight loss rate (%) = (weight before storage - weight after storage)/weight before storage × 100%.

(2) Good fruit rate: Fruits without disease, cold damage, mechanical damage and mold are considered as good fruits.

Good fruit rate (%) = (number of good fruits/total number of fruits) ×100%

(3) Hardness determination: A fruit hardness tester (Model FHT-1122, Guangzhou, China) was used for the determination. A peel with an area of approximately 0.25 cm² was removed at the equatorial position of the fruit and subsequently measured using a 0.79 mm diameter probe, with the hardness expressed in kg·cm^-^².

(4) Determination of soluble solids content (SSC,%): PAL-BX/ACID F5 type sugar-acid integrated machine (Atago, Japan) was used for the determination.

(5) Disease index statistics.

Calculations were made according to the grading of the area of surface disease incidence in kiwifruit, i.e., the area of brown spot on the fruit surface was counted as 0, 0-25%, 25%-50%, and > 50% as grades 0, 1, 2, and 3, respectively. The results were recorded and counted.


$$\begin{array}{c} \text{Disease index }\left(\%\right)=\\ \Sigma [\left(\text{Level}\times \text{number of fruits of the corresponding level}\right)/\\ \left(3\times \text{Total number of fruits}\right)]\times 100\% \end{array}$$

(6) Determination of color

The SC-10 colorimeter (3NH San Ench, China) was used to measure and record the data. The color difference formula was:


$$\text{Color difference }\text{Δ}\text{E}*\left(\text{NBS}\right)=\sqrt{{\left(\text{Δ}L^{*}\right)}^{2}+{\left(\text{Δ}{\text{α}}^{*}\right)}^{2}+{\left(\text{Δ}b^{*}\right)}^{2}}$$

In the formula: Δ L * represents the difference in brightness, Δα * represents the difference on the red/green axis, Δ b * represents the difference on the yellow/blue axis.

(7) Determination of TAC and VC content.

Determination of TAC (%) was carried out according to the method of Cao (Cao et al., 2007). Weigh 10g of the sample and transfer it to a 100 mL volumetric flask fix the volume with distilled water, let it stand for 30 min, and then filter it. Take 20.0 mL of filtrate into a triangular flask, add 2 drops of 1% phenolphthalein, titrate with calibrated sodium hydroxide solution, and titrate until the solution first shows pink and does not fade within half a minute as the endpoint (pH-8.1~8.3), record the amount of sodium hydroxide titrant, and then titrate the filtrate with distilled water instead of the filtrate as a blank control. The unit was expressed in percentage.

VC content (mg·100g^-1^) was determined with reference to the method of Li Y (Li et al., 2015). Accurately weigh 10 g of sample, add ethanedioic acid - EDTA solution, mashed or ground into a 100 mL volumetric flask filtration to absorb 10 mL of filtrate in a 50 mL volumetric flask, add 1 mL of metaphosphoric acid - acetic acid solution, 5% sulfuric acid 2 mL, shaking well, then add 4.00 mL of ammonium molybdate solution, with distilled water to 50 mL, 30°C water bath color development for 20 mins, removed after natural cooling, the absorbance value was measured at 705nm.

(8) Total RNA extraction, library preparation, and Illumina sequencing.

Genetic changes were determined in kiwifruit fruits treated with the PEHC. The experiment was divided into three groups, Group A was kiwifruit stored at 4°C on the 2nd day. Group B was the fruit at 4°C on the 30th day after PEHC treatment (at this time, the control group began to show signs of spoilage). Group C was control fruits stored at 4°C on the 30th day. After sampling the fruits of groups A, B, and C, they were quickly put into liquid nitrogen for quick freezing. Each group consists of 3 replicates, for a total of 9 samples. Samples were used for metabolite and total RNA extraction separately.

Total RNA was extracted using TRIzol Reagent (Plant RNA Purification Reagent for plant tissue). RNA degradation and contamination were assessed by electrophoresis on 1% agarose gels. After quality testing, the TruSeq RNA Sample Preparation Kit (Illumina, San Diego, CA) was used for library construction, following the main steps outlined in the manufacturer’s instruction manual. The constructed library was sequenced on an Illumina NovaSeq 6000 platform (2 × 150 bp read length). The sequencing process was performed by Shanghai Majorbio Bio-Pharm Technology Co., Ltd. (Shanghai, China). Raw sequences obtained from high-throughput sequencing were filtered and screened to generate high-quality clean reads for subsequent analysis.

Clean data from all samples were *de novo* assembled using Fastp (https://github.com/OpenGene/fastp), and the assembly results were optimized and evaluated. Subsequently, quality-controlled clean reads were aligned to the reference genome using HISAT2 (http://ccb.jhu.edu/software/hisat2/index.shtml). The clean reads of each sample were aligned to the reference transcript sequence using RSEM software. The number of reads mapped to each gene was then quantified for each sample. The expression level of each gene was calculated according to the transcripts per million reads (TPM) method. Differential gene expression analysis was conducted using the DESeq software, with screening criteria set at P-adjust ≤ 0.05 and |log_2_FC (fold change)| ≥ 1 (Choi et al., 2023). Differentially expressed genes (DEGs) were annotated and classified based on the Gene Ontology (GO) database. Additionally, the metabolic pathways associated with these DEGs were further analyzed using the Kyoto Encyclopedia of Genes and Genomes (KEGG) database.

(9) Analysis of metabolomics

The materials utilized for metabolomics analysis were identical to those used for transcriptomics sequencing. The sequencing analysis was conducted by Shanghai Majorbio Bio-Pharm Technology Co., Ltd. (Shanghai, China). For metabolite extraction, 50 mg of the solid sample was transferred into a 2 mL centrifuge tube containing a 6 mm grinding bead. Subsequently, 400 μL of extraction solution (methanol: water = 4:1, v/v), containing 0.02 mg/mL of internal standard (L-2-chlorophenylalanine), was added to the tube to facilitate metabolite extraction. The sample was ground using a Wonbio-96c (Shanghai Wanbai Biotechnology Co., Ltd.) frozen tissue grinder for 6 mins (-10°C, 50 Hz), followed by low-temperature ultrasonic extraction for 30 mins (5°C, 40 kHz). Place the sample at -20°C for 30 mins, centrifuge for 15 mins (4°C, 13,000 g), and transfer the supernatant to an injection bottle for LC-MS/MS analysis. The mass spectrometry data obtained were processed using the software Progenesis QI (Waters Corporation, Milford, USA), including chromatographic peak integration and correction. Substance identification was performed based on secondary mass spectrometry (MS/MS) information using public databases (HMDB and Metlin) and Majorbio’s in-house database. Quantitative analysis was conducted using the Majorbio Cloud platform (cloud.majorbio.com). For statistical analysis, principal component analysis (PCA) and orthogonal partial least squares discriminant analysis (OPLS-DA) were carried out using the ropls package (Version 1.6.2) in R language. Differential metabolites were screened based on a variable importance in projection (VIP) score of ≥ 1 and a significance threshold of P< 0.05 (Wu et al., 2022). Finally, the Kyoto Encyclopedia of Genes and Genomes (KEGG) database was utilized to annotate the differential metabolites and identify the associated metabolic pathways.

(10) qRT-PCR analysis: To validate the RNA-seq results, three ethylene-related genes were selected for qRT-PCR analysis. The experimental determination was conducted by Shanghai Majorbio Bio-pharm Technology Co., Ltd. (Shanghai, China). The RNA extracted as described in step (8) was subjected to quality assessment and concentration measurement using 1.0% agarose gel electrophoresis and micro-UV spectrophotometry (Thermo NanoDrop 2,000, Thermo Fisher Scientific, Waltham, MA, USA), respectively. Approximately 1 μg of total RNA was used for cDNA synthesis using the RevertAid first-strand cDNA synthesis kit (Thermo Fisher Scientific, Waltham, MA, USA). Three biological replicates were set up for each sample. The primers were designed using the software Primer Premier 6 and are listed in **Table 3**. A total of 0.2 mL PCR tubes were prepared, along with 10 μL of 2×Universal Blue SYBR Green qPCR Master Mix, 1.0 μL of 10 μM gene primer, 1.0 μL of 10 μM reverse transcription product, and 3 tubes of each reverse transcription product, with the addition of nuclease-free water. Nuclease-free water was added to a final volume of 20 μL, and three tubes were prepared for each reverse transcription product. The PCR reaction program consisted of the following stages: stage 1: pre-denaturation at 95°C for 30 secs; stage 2: denaturation at 95°C for 15 secs, annealing/extension at 60°C for 30 secs, and repetition for 45 cycles; stage 3: solubilization curve. Gene expression was calculated using the 2^-ΔΔCt^ method, with GAPDH as the internal reference.

**Table 3:** Primer list.

Serial number	Primer Name	Sequence (5’→3’)
1	Acc24995-F	CGGTGGCATCATCCTCCTCTT
2	Acc24995-R	CACGGTGCATCACGCTCTTG
3	Acc20538-F	CCGACGCTGGTGGAATCATC
4	Acc20538-R	TCACGCTCTTGTACTTGCCATT
5	Acc17490-F	GCTGCTCAAGGATGACAAATGG
6	Acc17490-R	GGTTGTAGAACGAGGCTATGGA
7	GAPDH-F	ACACTCCATCACTGCGACA
8	GAPDH-R	CACCTTGCCAACAGCCTTA

(11) ACC content determination

ACC content was determined by high-performance liquid chromatography, and sequencing analysis was performed by Shanghai Majorbio Bio-pharm Technology Co., Ltd. (Shanghai, China). Take out the sample at -80 °C, grind it with liquid nitrogen, weigh 0.1g of the sample, and add 1170uL of acetonitrile formic acid solution (80:19:1, v/v/v), 10uL of ISMix-A, 20uL of ISMix-B, vortex the mixture for the 60s, low-temperature dark ultrasound for 25 mins, and let it stand overnight at -20 °C, 14000 rpm, centrifuge at 4 °C for 20 mins, take 900 µL of the supernatant through an Ostro 25 mg 96-well lipid removal plate under positive pressure. Then add 200uL of acetonitrile-water formic acid solution (80:19:1, v/v/v) for elution once, dry the filtrate with liquid nitrogen, and store the sample at -80 °C. The samples were separated using an ultra-high performance liquid chromatography (1,290 Infinity UPLC, Agilent) system. Mass spectrometry was analyzed using a 5,500 QTRAP mass spectrometer (5,500 QTRAP, SCIEX) in both positive and negative ion modes. Use Multiquant 3.0.2 software to extract chromatographic peak area and retention time. The use of standard samples of the target substance to correct retention time and metabolite identification was subsequently conducted.

### 2.3 Data analysis

Data were counted using WPS Office, statistically analyzed using Prism 10.1.2 and Origin Pro 2022 software, and correlation heatmaps were produced using Tbtools. Different *’s in the graphs indicate comparisons between the experimental treatments, where * represents p<0.05, ** represents p<0.01, *** represents p<0.001, and **** represents p<0.0001.

## 3 Results and analysis

### 3.1 Results of the one-way test

#### 3.1.1 Effect of BRs concentration on the storage quality of postharvest kiwifruit

**Figure 1:**
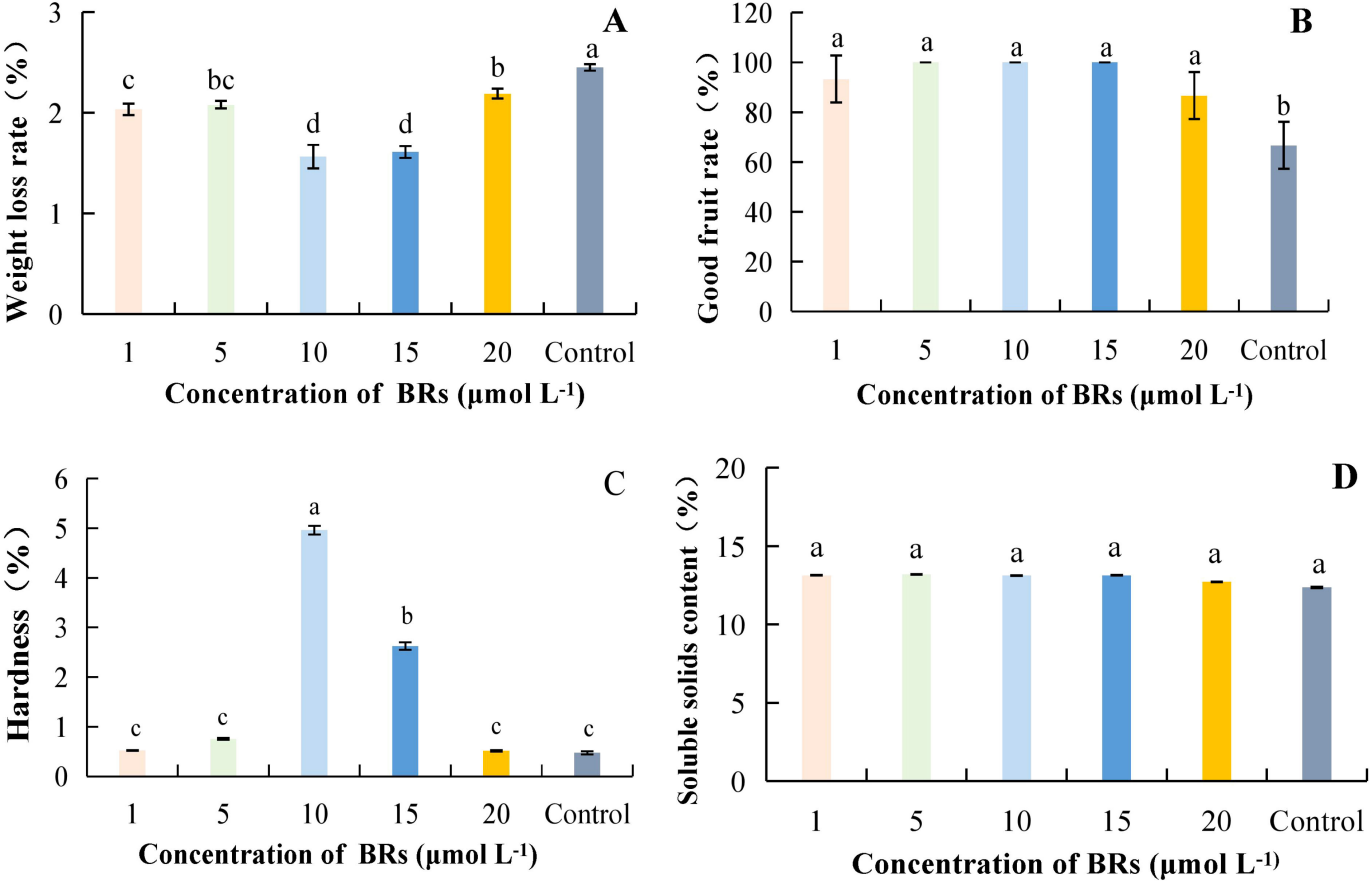
The effect of different concentrations of brassinolide on the quality of postharvest kiwifruit. BRs, brassinolide. **(A)** weight loss rate. **(B)** good fruit rate. **(C)** hardness. **(D)** soluble solids content (SSC). Different lower case letters represent significant differences (p<0.05).

As the concentration of BRs increased, the overall weight loss rate of kiwifruit showed a decreasing and then increasing trend; the control treatment was significantly higher than the treatment groups (**Figure 1A**). Among them, the weight loss rate at a concentration of 15 μmol·L^-1^ was 56.72% lower than that of the control. The good fruiting rate of kiwifruit treated with BRs was higher than that of the power in all cases, and the difference was significant in the treatment group compared with the control (p< 0.05). In contrast, the difference between treatments was not substantial (**Figure 1B**). The highest fruit hardness was observed at a concentration of 10 μmol·L^-1^ of BRs, which was 943.48% higher than that of the control (**Figure 1C**). The differences between the BRs at different concentrations were not significant compared with the control; at a concentration of 10 μmol·L^-1^, the SSCs of the SA treatment were consistent with the comparison (**Figure 1D**). Taken together, it can be concluded that the successive concentrations of BRs ranging from 5 to 15 μmol·L^-1^ resulted in reduced weight loss rate and higher good fruit rate and hardness.

#### 3.1.2 Effect of MT concentration on storage quality of postharvest kiwifruit fruit

**Figure 2:**
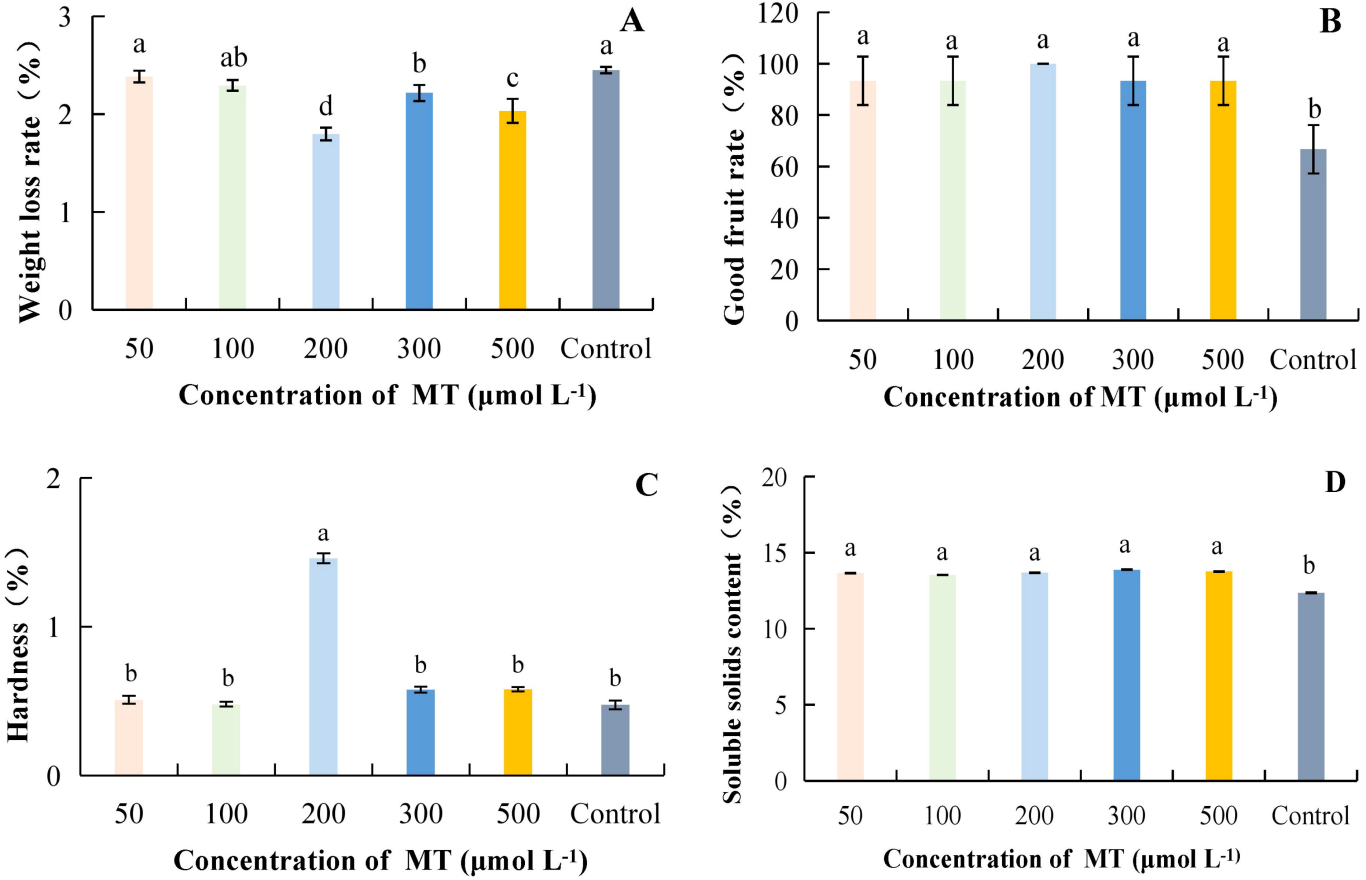
The effect of different concentrations of MT on the storage quality of kiwifruit. MT, Melatonin. **(A)** weight loss rate. **(B)** good fruit rate. **(C)** hardness. **(D)** soluble solids content (SSC). Different lower case letters represent significant differences (p<0.05).

As MT concentration increased, weight loss rate was lowest at 200 μmol·L^-1^ at 1.80%, which was 36.36% lower than the control (**Figure 2A**). At a MT concentration of 200 μmol·L^-1^, the rate of good fruits was highest at 100%; it increased by 50.00% compared to the control, and the difference was significant. However, the differences between treatments with different MT concentrations were not significant (**Figure 2B**). Hardness was highest at 200 μmol·L^-1^, with a value of 1.46 kg·cm^-2^, and it differed significantly from other treatments, being 207.01% higher than the control (**Figure 2C**). The SSC was highest at a MT concentration of 300 μmol·L^-1^, which was 12.30% higher than the control and significantly different from it (**Figure 2D**). Taken together, it can be concluded that MT at successive concentrations of 200 to 500 μmol·L^-1^ resulted in lower weight loss, a higher good fruit rate, and increased hardness.

#### 3.1.3 Effect of MeJA concentration on storage quality of postharvest kiwifruit fruit

**Figure 3:**
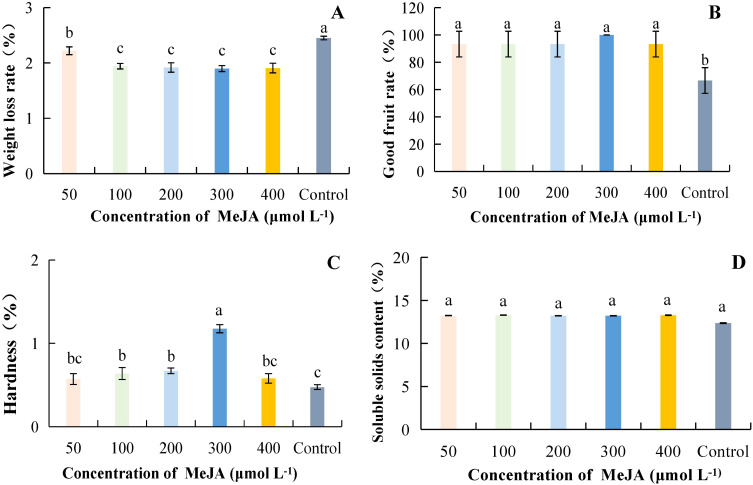
Effect of MeJA concentration on storage quality of kiwifruit fruit. MeJA, Methyl jasmonate. **(A)** weight loss rate. **(B)** good fruit rate. **(C)** hardness. **(D)** soluble solids content (SSC). Different lower case letters represent significant differences (p<0.05).

As the concentration of MeJA increased, weight loss was lowest at 300 μmol·L^-1^, with a percentage of 1.90%, which was 22.59% lower than the control, and the difference was significant when compared to the control (**Figure 3A**). The highest percentage of good fruits was 100% at a MeJA concentration of 300 μmol·L^-1^; it increased by 50.00% compared with the control, and the difference was significant. However, the differences between treatments with different concentrations were not significant (**Figure 3B**). Hardness was highest at 300 μmol·L^-1^, with a value of 1.18 kg·cm^-2^, and it was significantly different from other treatments, being 147.41% higher than the control (**Figure 3C**). Although the different concentrations of MeJA treatments increased SSC compared to the control, the differences between treatments were not significant (**Figure 3D**). Taken together, it can be concluded that weight loss was reduced and good fruit rate and hardness were highest at successive concentrations of MeJA ranging from 200 to 400 μmol·L^-1^.

#### 3.1.4 Effect of SA concentration on storage quality of postharvest kiwifruit

**Figure 4:**
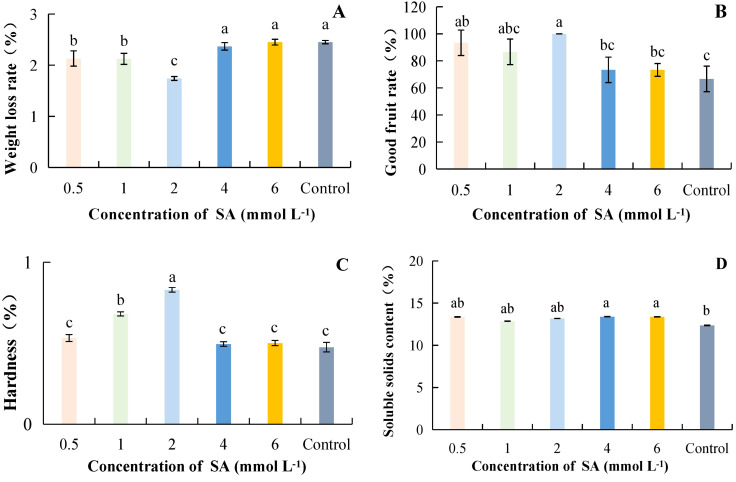
The effect of salicylic acid concentration on the storage quality of kiwifruit. SA, Salicylic acid. **(A)** weight loss rate. **(B)** good fruit rate. **(C)** hardness. **(D)** soluble solids content (SSC). Different lower case letters represent significant differences (p<0.05).

As the concentration of SA increased, weight loss was lowest at 2 mmol·L^-1^ at 1.74%, which was 40.80% lower than the control and significantly different from the control (**Figure 4A**). The highest good fruit rate was 100% at SA concentration of 2 mmol·L^-1^; it increased by 50.00% compared to control (**Figure 4B**). Hardness was highest at 2 mmol·L^-1^ at 0.83 kg·cm^-2^ under different concentrations of SA treatments; the difference was significant as compared to other treatments and was 74.61% higher as compared to control (**Figure 4C**). Different concentrations of SA treatments increased the SSC compared to the control. However, the differences between SA treatments and the control were not significant at concentrations ranging from 0.5 to 2 mmol·L^-1^ (**Figure 4D**). Overall, it can be concluded that the successive concentrations of SA ranging from 0.5 to 2 mmol·L^-1^ resulted in lower weight loss and a higher good fruit rate, and hardness.

#### 3.1.5 Heat map analysis

(1) Inter group difference heatmap analysis.

**Figure 5:**
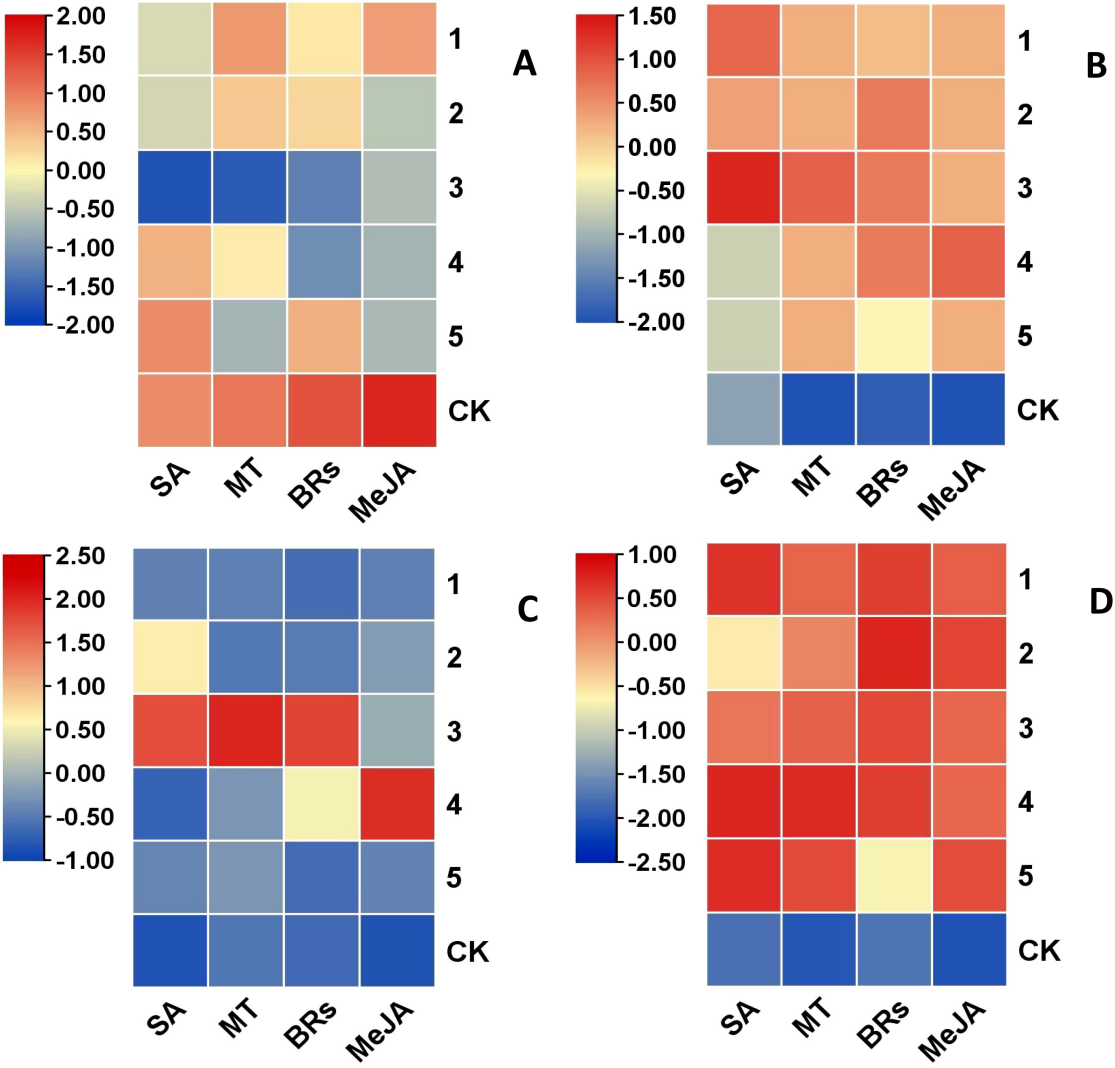
Heat map of differences between treatments for the same indicator. **(A)** weight loss rate. **(B)** good fruit rate. **(C)** hardness. **(D)** soluble solids content (SSC). 1, 2, 3, 4 and 5 in the graph represent the concentration levels of different hormones, respectively (see **Table 1**).

One-way analysis of the four hormone concentrations yielded heat plots of intergroup differences in weight loss rate, good fruiting rate, hardness, and SSC in kiwifruit fruit (**Figures 5A–D**); the differences in fruit good fruit rate and SSC were not significant under different hormone concentrations (all heat plots showed red color). Differences in weight loss rate under different concentrations of hormone treatments were general (SA, MT, and BRs had significantly lower fruit weight loss rate at the three gradient concentrations than at the other treatment concentrations). The greatest differences in hardness were observed under different concentrations of hormone treatments (SA, MT, and BRs at three gradient concentrations, and MeJA at four gradient concentrations, and fruit hardness was significantly higher than hardness at other treatment concentrations). Therefore, hardness was chosen as the measurement index in the orthogonal experiment.

(2) Heat map analysis of correlation between fruit indicators under different concentrations of hormone treatments.

**Figure 6:**
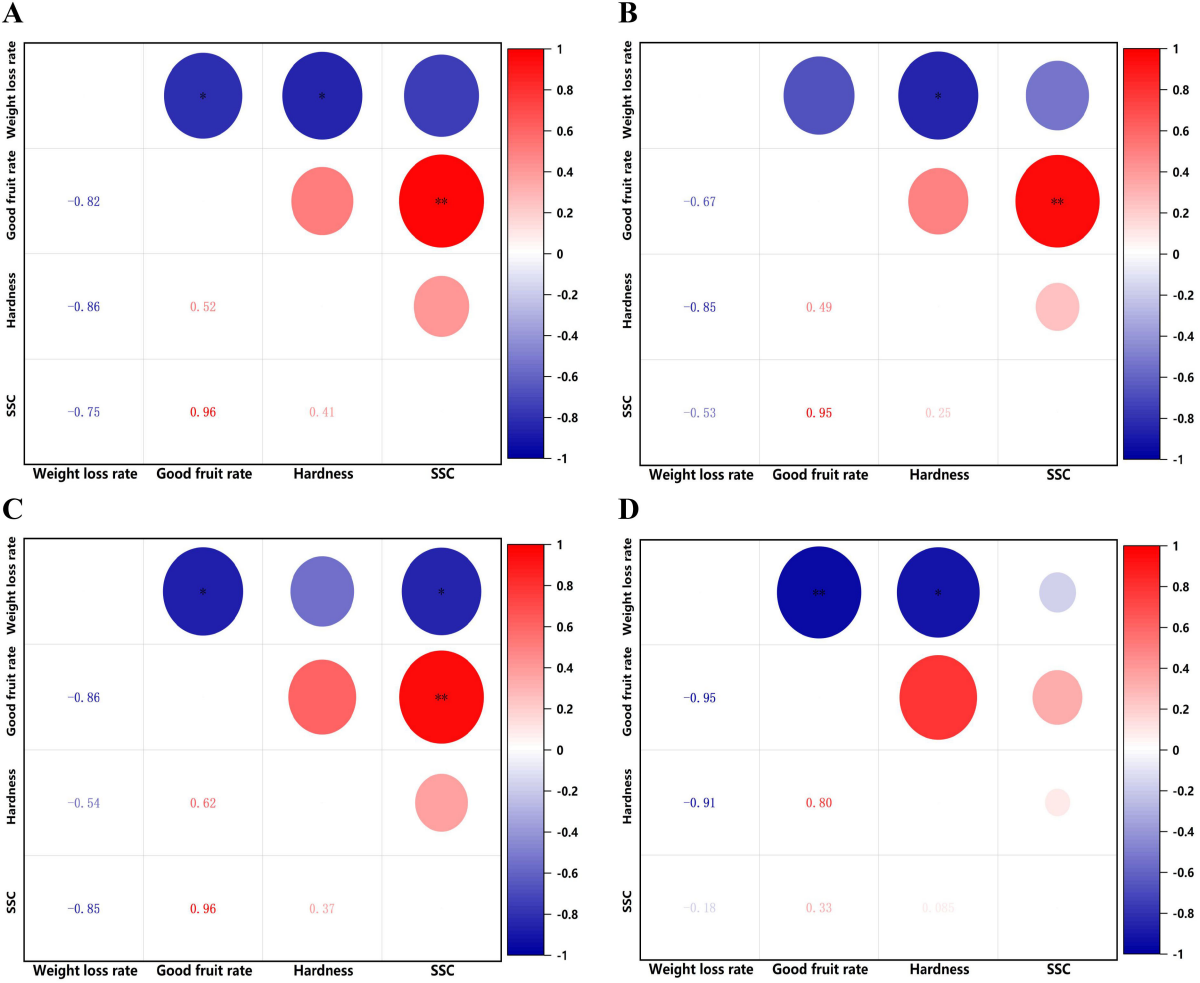
Heat map of correlation between different indicators for each treatment. **(A)** Brassinolide. **(B)** Melatonin. **(C)** Methyl jasmonate. **(D)** Salicylic acid. Red indicates a positive correlation, blue indicates a negative correlation, The darker the color, the more significant the correlation. **P< 0.01. *P< 0.05.

Under different concentrations of hormone treatments, all treatments showed a negative correlation between weight loss rate and good fruit rate, hardness, and SSC, a positive correlation between good fruit rate and SSC, hardness, and a positive correlation between hardness and SSC (**Figures 6A–D**).

### 3.2 Orthogonal test results

According to the orthogonal test design table, three optimal levels of four hormones, BRs, MT, MeJA, and SA, were used in orthogonal tests in combination with the results of the one-way test (**Table 4**).

**Table 4:** Orthogonal test results and analysis.

Factor					
Level	Brassinolide(μmol·L^-1^)	Melatonin(μmol·L^-1^)	Methyl jasmonate(μmol·L^-1^)	Salicylic acid(mmol·L^-1^)	Fruit hardness(kg cm^-2^)
	A	B	C	D	
1	5	200	100	0.5	2.011 ± 0.23DE
2	5	300	200	1	1.709 ± 0.31E
3	5	500	300	2	1.787 ± 0.32E
4	10	200	200	2	5.847 ± 0.19A
5	10	300	300	0.5	4.924 ± 0.20B
6	10	500	100	1	4.536 ± 0.23B
7	15	200	300	1	3.732 ± 0.25C
8	15	300	100	2	2.616 ± 0.26D
9	15	500	200	0.5	2.149 ± 0.28DE
K1	1.836	3.863	3.054	3.028	
K2	5.102	3.083	3.235	3.326	
K3	2.832	2.824	3.481	3.417	
R	3.266	1.039	0.427	0.389	
Optimum condition	A2	B1	C3	D3	

A is Brassinolide, B is Methyl jasmonate, C is Methyl jasmonate, D is Salicylic acid.

The optimal process formulation for kiwifruit phytohormone synergistic treatment was A_2_B_1_C_3_D_3_ as shown by the K. The extremely different results indicated that the primary and secondary factors affecting the hardness of kiwifruit were A > B > C > D, i.e., BRs concentration > MT concentration > MeJA concentration > SA concentration (**Table 4**). After obtaining the optimal formulation process combination from the orthogonal test, we conducted a validation test to confirm its effectiveness. The hardness of kiwifruit fruit was measured to be 5.97 kg·cm^-^², and the results of the orthogonal test were found to be accurate and reliable. The optimal hormone formulation combination i.e., BRs concentration of 10 μmol·L^-1^, MT concentration of 200 μmol·L^-1^, MeJA concentration of 300 μmol·L^-1^, and SA concentration of 2 mmol·L^-1^.

### 3.3 Effect of plant endogenous hormones complex (PEHC) on the appearance quality of kiwifruit fruits

#### 3.3.1 Effect of PEHC on weight loss rate and good fruit rate of postharvest kiwifruit

**Figure 7:**
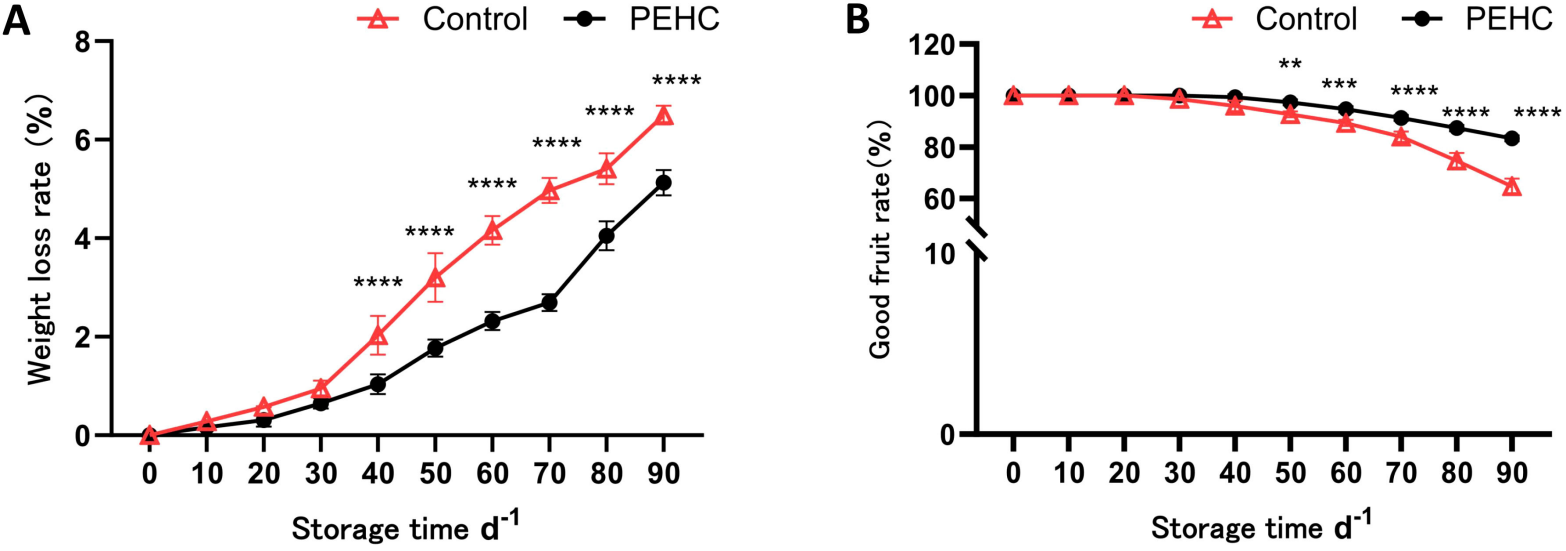
Effect of the PEHC on weight loss rate and good fruit rate of postharvest kiwifruit. PEHC, Plant endogenous hormones complex. **(A)** Weight loss rate. **(B)** Good fruit rate. ****P<0.0001. ***P< 0.001. **P< 0.01.

The rate of weight loss increased with increasing storage time (**Figure 7A**). After 30 d, fruit weight loss began to increase substantially in the control and treated groups. The largest difference in weight loss rate between the PEHC and the control was 2.27% at 70 d, when the weight loss rates were 2.69% and 4.97%, respectively. At 90d, the fruit weight loss rate was 5.17% and 6.50% for the PEHC and control, respectively, 21.16% lower than the control. After 40d of storage, there was a significant difference in weight loss rate between the PEHC and the control at P<0.01. The PEHC can reduce the weight loss rate of the fruits at the late storage stage.

The good fruit rate of kiwifruit began to decline gradually after 30 d of storage, in which the good fruit rate of the PEHC was higher than that of the control. At 90 d of storage, the difference between the two treatments was the largest, with 83.33% and 64.67% for the PEHC and the control, respectively. The PEHC increased the good fruit rate by 28.87% compared with the control (**Figure 7B**). After 50d of storage, there was a highly significant difference (P<0.01) between the PEHC and the control in terms of good fruit rate, with the PEHC of kiwifruit having a higher good fruit rate than the control.

#### 3.3.2 Effect of the PEHC on postharvest kiwifruit hardness and disease index

**Figure 8:**
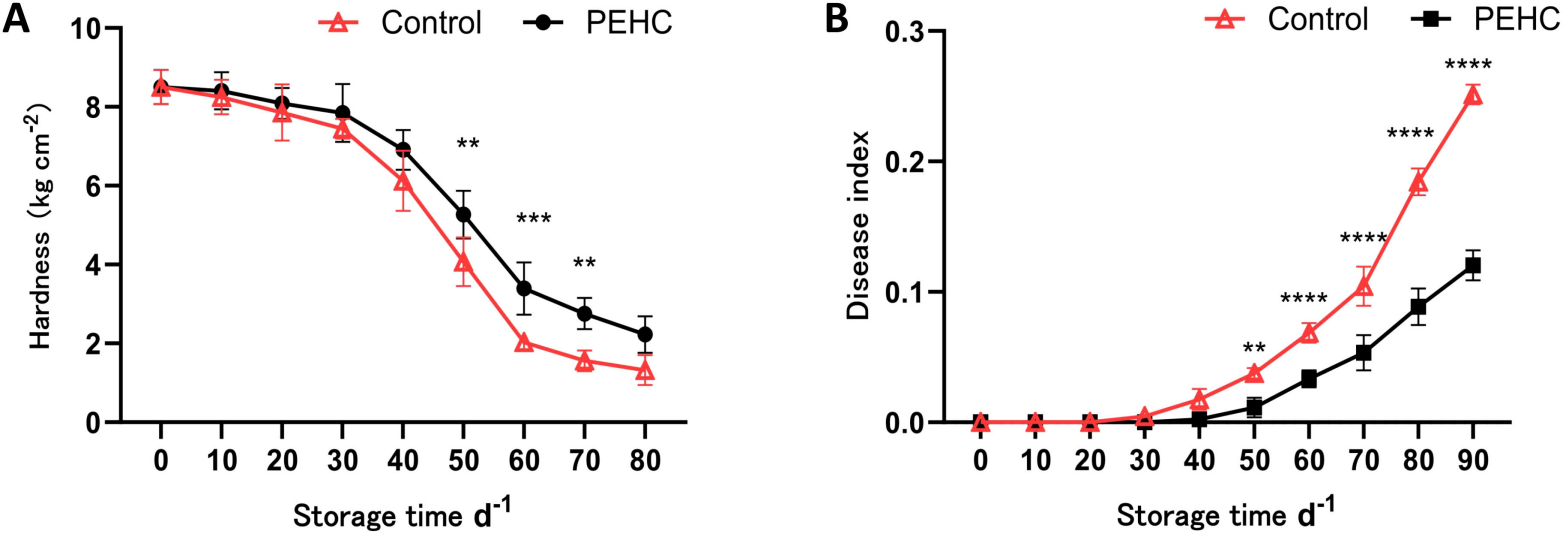
Effect of the PEHC on postharvest kiwifruit hardness and disease index. PEHC, Plant endogenous hormones complex. **(A)** Hardness. **(B)** Disease index. ****P<0.0001. ***P< 0.001. **P< 0.01.

The hardness of kiwifruit generally decreased with storage time (**Figure 8A**). After 30 d of storage, fruit hardness began to show a substantial decline; at 60 d of storage, the PEHC was 1.36 kg·cm^-2^ higher than that of the control; at 80 d of storage, the hardness of the PEHC and the control decreased to 2.23 kg·cm^-2^ and 1.32 kg·cm^-2^, respectively, and that of the PEHC was 0.91 kg·cm^-2^ higher than that of the control. After 50d of storage, the fruit hardness of the PEHC showed a highly significant difference (P<0.01) with the control. It can be seen that the PEHC had the effect of delaying postharvest fruit softening and could better maintain fruit quality.

After 30 d of storage, the fruit disease index of kiwifruit began to show a gradually increasing trend (**Figure 8B**). The largest difference between the two groups occurred at 90 days of storage, with the fruit disease index for the PEHC and the control being 0.12 and 0.25, respectively. The fruit disease index of the PEHC was reduced by 0.13 compared to the control. The fruit disease index of the PEHC and the control showed highly significant differences from the 50th to the 90th day of storage (P<0.01), indicating that the PEHC treatment was effective in reducing the disease index of kiwifruit during storage.

### 3.4 Effect of the PEHC on the intrinsic quality of kiwifruit

#### 3.4.1 Effects of the PEHC on SSC and color of postharvest kiwifruit

**Figure 9:**
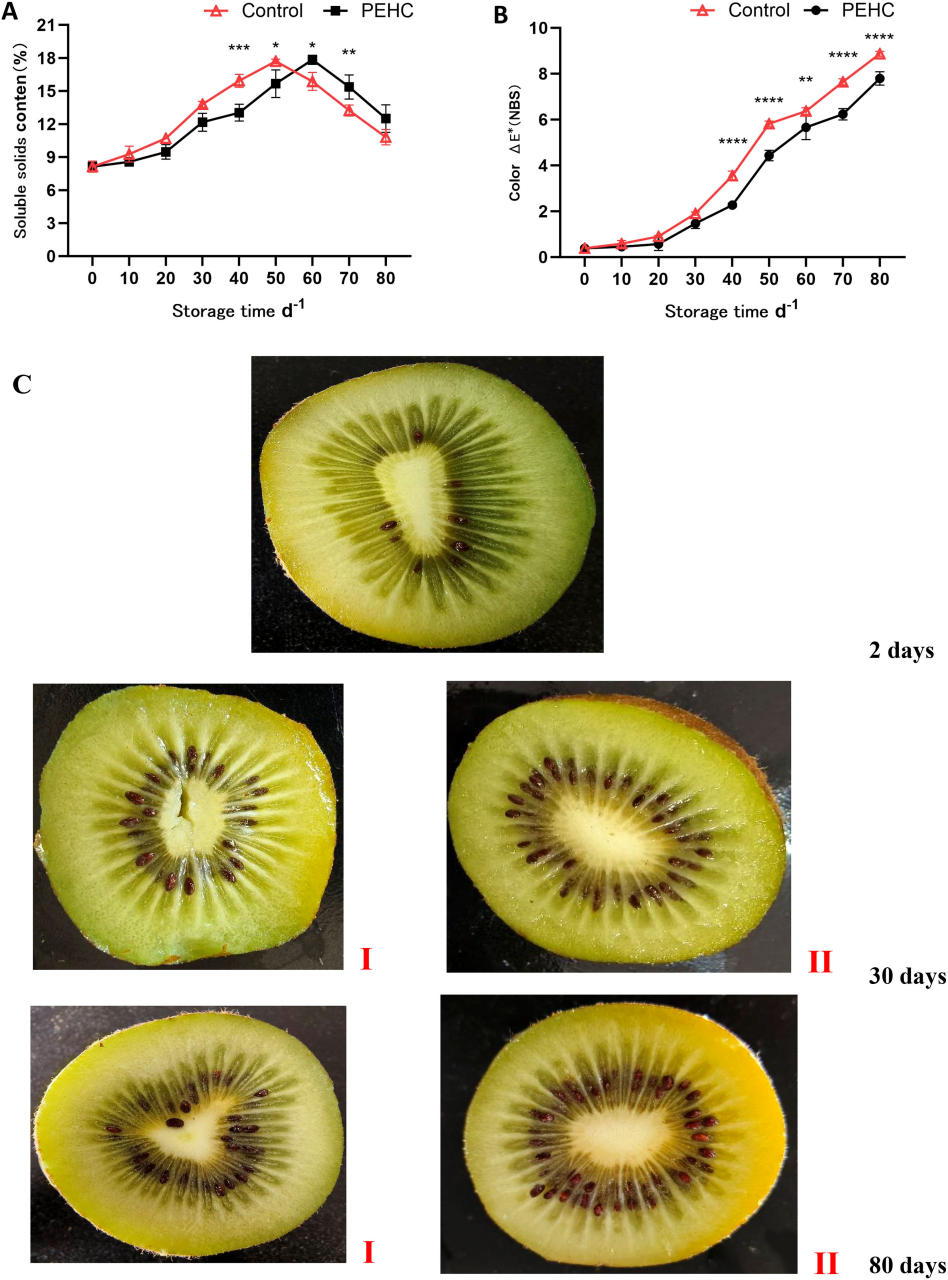
Effect of the PEHC on soluble solids content and color of kiwifruit. PEHC, Plant endogenous hormones complex. **(A)** Soluble solids content (SSC). **(B)** Color. **(C)** comparison of fruit flesh color between PEHC group (I) and control group (II). ****P<0.0001. ***P< 0.001. **P< 0.01. *P< 0.05.

From 0 to 50 days of storage, the SSC of kiwifruit showed a gradually increasing trend, and the SSC of the control was higher than that of the PEHC (**Figure 9A**). At 50d, the SSC of the control started to show a decreasing trend, while that of the PEHC started to show a decreasing trend after 60d. It can be seen that the PEHC was beneficial for delaying the accumulation of SSC content in the early stage. At day 80, the SSC of the PEHC was higher than that of the control, but the difference was not significant.

The color difference between the two groups of kiwifruit was not significant during the initial 0 to 30 days of storage (**Figures 9B, C**). After 40 d, a highly significant difference between the two treatments was observed (P<0.01). At 70d of storage, the PEHC had an 18.47% lower fruit color than the control. At 80d, the color between kiwifruit in the PEHC and control was 7.79 and 8.87, respectively, which was 12.17% lower in the PEHC compared to the control. With the extension of storage time, the color between the two groups of kiwifruit was gradually obvious, and the color was gradually darker and yellowish brown. The PEHC affected the color change of kiwifruit fruits to some extent.

#### 3.4.2 Effect of the PEHC on TAC and VC content of postharvest kiwifruit

**Figure 10:**
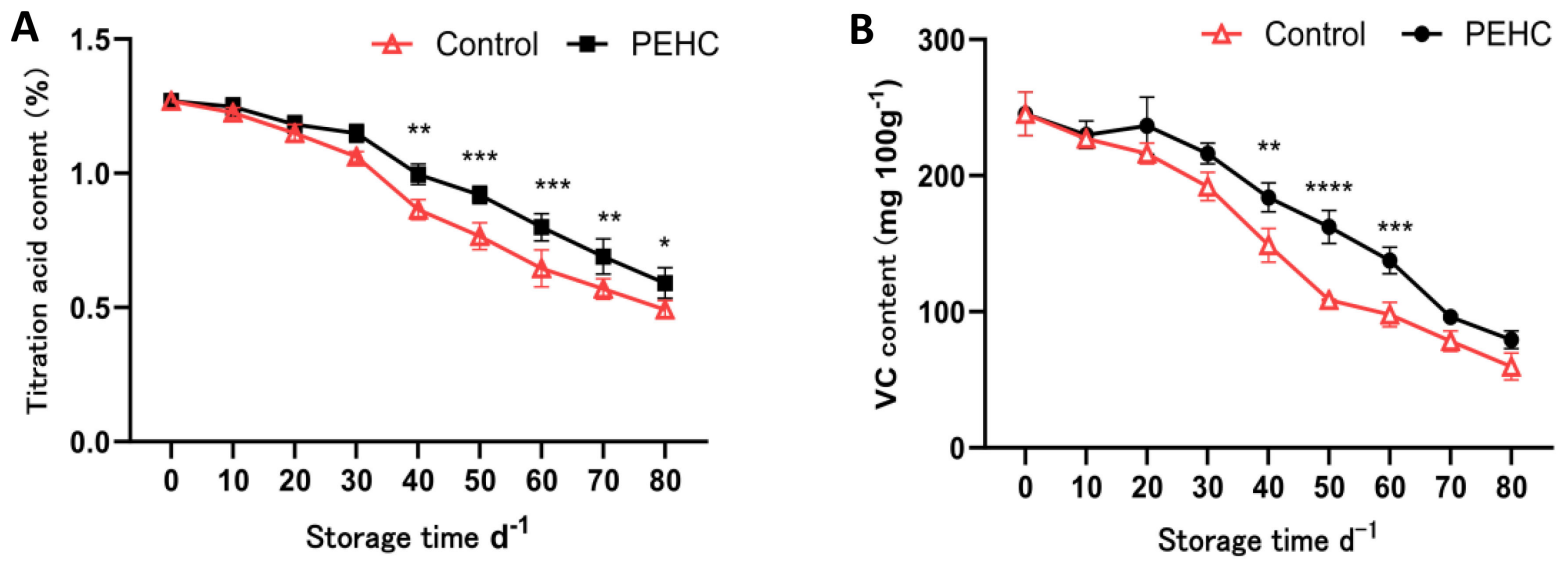
Effect of the PEHC on TAC and VC content of kiwifruit. PEHC, Plant endogenous hormones complex. **(A)** Titration acid content (TAC). **(B)** VC content. ****P<0.0001. ***P< 0.001. **P< 0.01. *P< 0.05.

The TAC of the two treatments showed an overall decreasing trend with the extension of storage time; after 40 d of storage, the PEHC had 15.19% higher TAC than the control (**Figure 10A**). At 80 d, the TAC of the fruit was 0.59% and 0.49% for the PEHC and control, respectively, and the PEHC was 20.00% higher than the control. At 40d, the PEHC was significantly different (P<0.05) from the control.

At 10 to 20 d, the VC content of PEHC kiwifruit increased slightly (**Figure 10B**), and at 50 d the VC content of the PEHC fruits was higher than that of the control by 53.71 mg·100 g^-1^. At 80 d of storage, the fruit VC content of PEHC and control decreased to 79.31 mg·100 g^-1^ and 59.74 mg·100 g^-1^, respectively, and the fruit VC content of PEHC was higher than that of the control by 19.57 mg·100 g^-1^. At 40-60 d, the PEHC was highly significantly different from the control (p<0.01), indicating that the PEHC of kiwifruit was beneficial in delaying the reduction of fruit VC content.

### 3.5 Heat map analysis of fruit quality indicators

**Figure 11:**
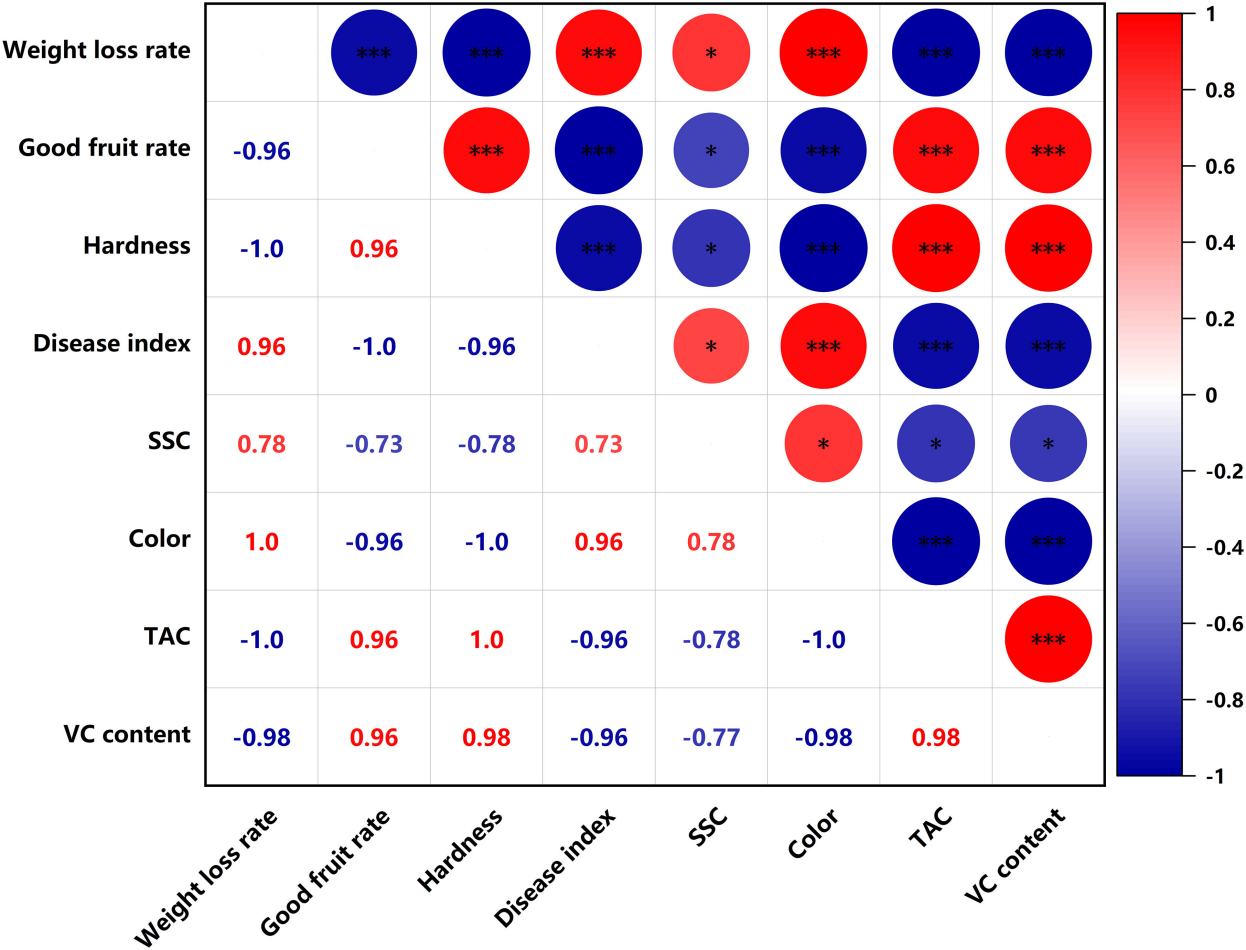
Heat map of correlation of quality indicators in hormone synergistic treatment groups. ***P< 0.001, *P< 0.05.

**Figure 12:**
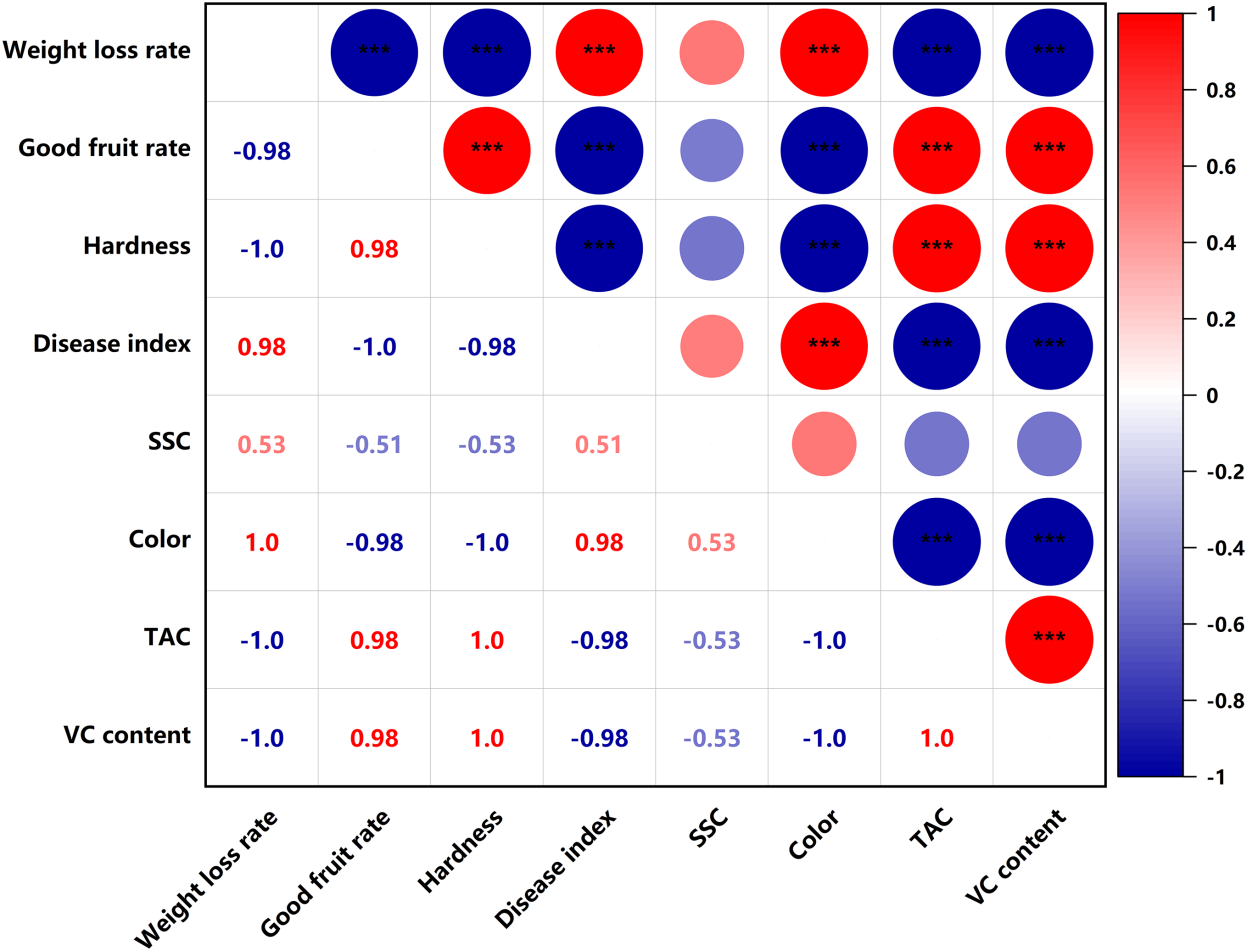
Heat map of correlation of quality indicators in the control. ***P< 0.001. **P< 0.01.

SSC were significantly negatively correlated (p<0.05) with titratable acid content and VC content in the PEHC (**Figure 11**). In PEHC and control, weight loss rate was highly significantly negatively correlated with good fruit rate, TAC and VC content, and highly significantly positively correlated with disease index and color (p<0.001); fruit hardness was highly significantly positively correlated with TAC and VC content; good fruit rate was highly significantly negatively correlated with weight loss rate, disease index and color, and highly significantly positively correlated with fruit hardness, TAC and VC content (p<0.001, **Figures 11**, **12**).

### 3.6 Differential metabolite classification and KEGG enrichment analysis

**Figure 13:**
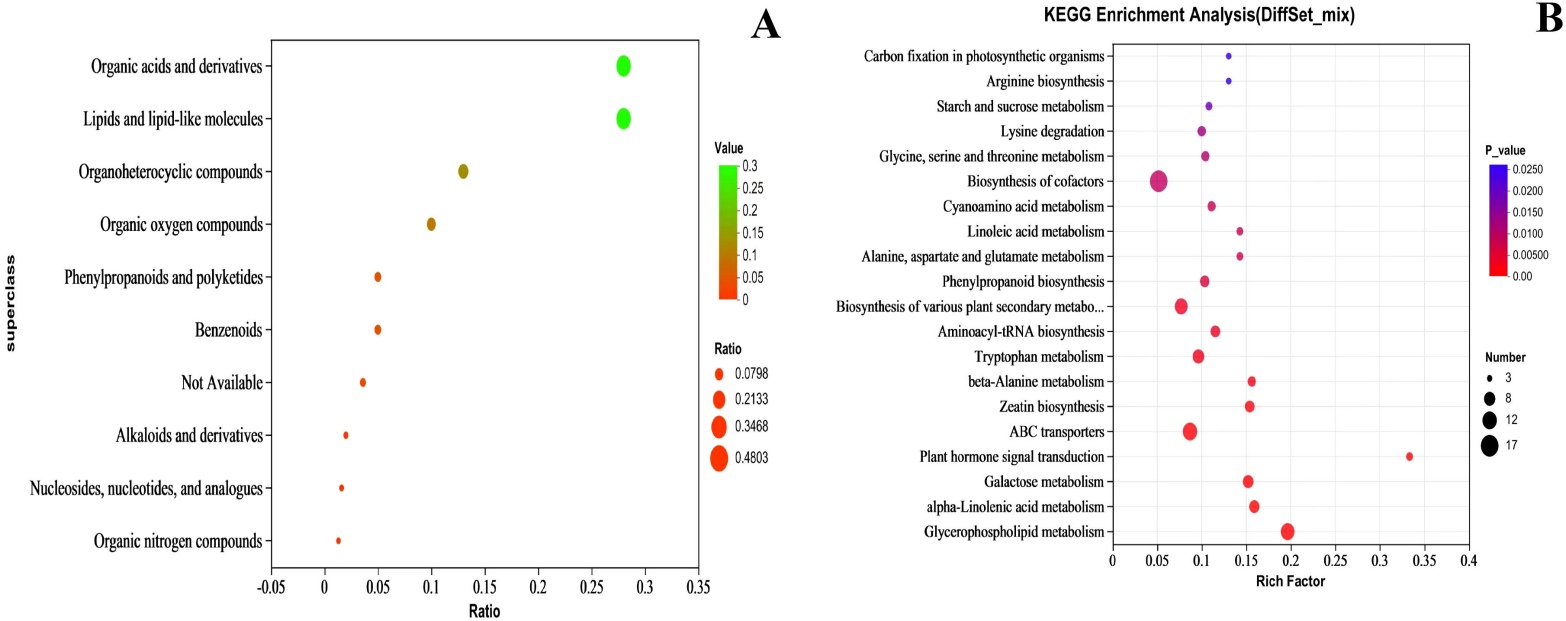
Compound classification **(A)** and KEGG **(B)** KEGG enrichment analysis of metabolites. In **(A)** the horizontal coordinate is Ratio, indicating the relative proportion of metabolite quantities in each classification, and the vertical coordinate is for each classification, with both bubble color and size indicating the Ratio value. In **(B)** the horizontal coordinate is the enrichment rate and the vertical coordinate is the KEGG pathway. The size of the bubbles in the graph represents how much of the pathway is enriched to compounds in the metabolic set, and the color of the bubbles indicates the size of the p-value for different enrichment significance.

According to the HMDB superclass classification, the compounds were divided into 10 categories. The top 5 categories were Lipids and lipid-like molecules, Organic acids and derivatives, Organoheterocyclic compounds, Organic oxygen compounds, and Phenylpropanoids and polyketides, containing 125, 125, 61, 44, and 22 substances, respectively (**Figure 13A**). The content of these substances was closely related to the quality of the fruit flesh.

The top 20 KEGG metabolic pathways are shown in **Figure 13B**. Among them, Plant hormone signal transduction is related to plant hormone metabolism, Starch and sucrose metabolism is associated with sugar metabolism, and Phenylpropanoid biosynthesis is linked to plant pigment metabolism.

The biosynthesis of various plant secondary metabolites is a significant source of fruit aroma components, particularly terpenoids and esters. Glycerophospholipid metabolism, alpha-linolenic acid metabolism, and Linoleic acid metabolism are related to fatty acid metabolism, while alanine, aspartate, and glutamate metabolism and Glycine, serine and threonine metabolism are associated with amino acid.

### 3.7 Categorical analysis of differential metabolites

**Figure 14:**
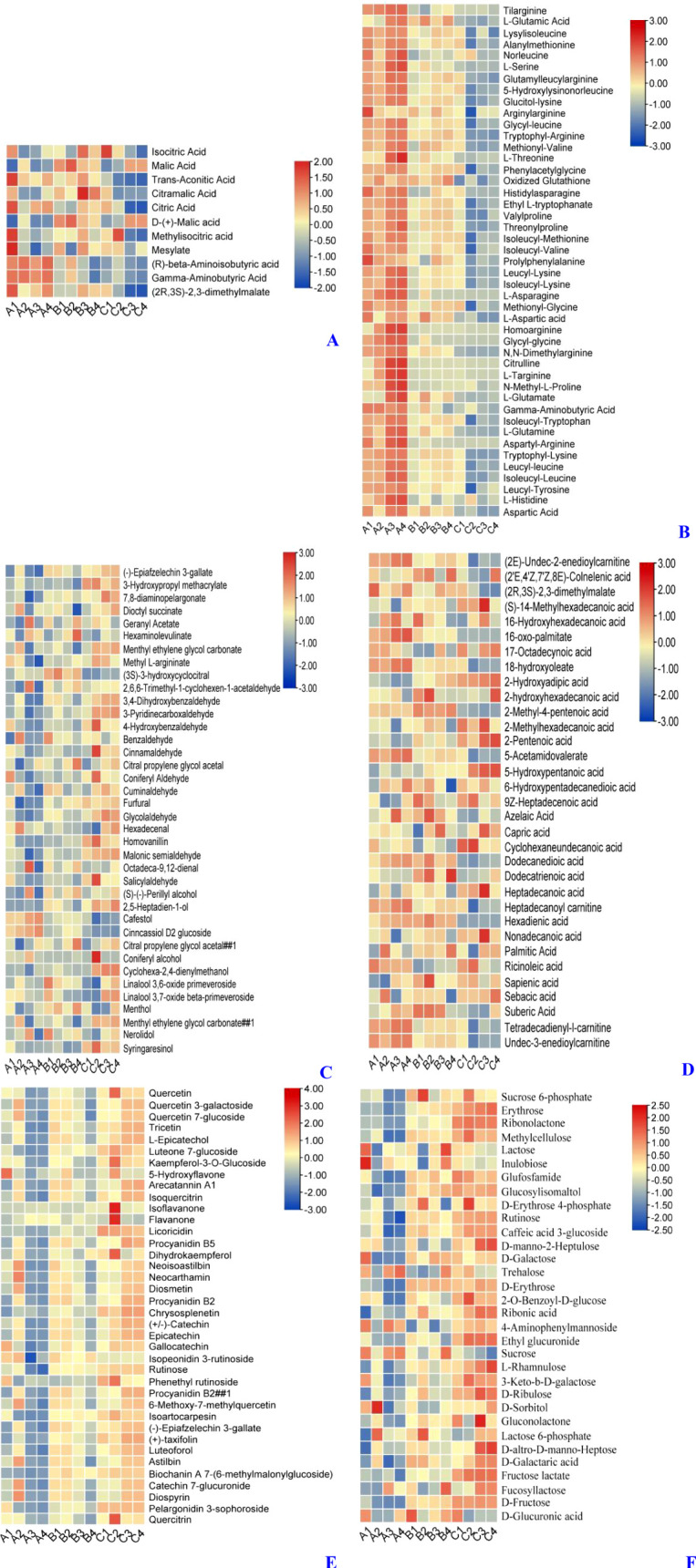
Heat map of differential metabolites (DAMs) between treatments. A1, A2, and A3 were treatment group A (kiwifruit stored at 4 °C for the second day). B1, B2, and B3 were treatment group B (kiwifruit stored at 4 °C for 30 days after PEHC treatment, at which point the control group begins to show signs of spoilage). C1, C2, and C3 were treatment group C (kiwifruit stored at 4 ° C for 30 days). **(A)** Acids. **(B)** Amino acids. **(C)** aroma. **(D)** fatty acids. **(E)** pigments or phenolics. **(F)** Soluble sugar.

To further investigate the effect of PEHC on kiwifruit storage quality, we used metabolomics to study the metabolites in the pulp of each treated kiwifruit (**Figure 14**). Combining total ion current (TIC) and multiple reaction monitoring (MRM) assays, we finalized 1,552 metabolites (1,075 in positive ion mode and 477 in negative ion mode). Meanwhile, we focused on the analysis of (**Figure 14A**) acids, (**Figure 14B**) amino acids, (**Figure 14C**) odors, (**Figure 14D**) oils (**Figure 14E**) coloring, and (**Figure 14F**) soluble sugars, which affect the quality of fruits. The results showed that 11 acids (**Figure 14A**) were identified. Overall, the acid content in fruits from Group C was lower, while the acid content in fruits from Groups A and B was higher. This suggests that PEHC delayed the reduction in acidity, with results similar to those shown in **Figure 10A**. Forty-five amino acids (**Figure 14B**) were identified, and the content of amino acids was relatively highest in group A, followed by group B, and lowest in group C, indicating that the PEHC could delay the decrease of amino acids.

Thirty-eight aromas (**Figure 14C**) were identified. Overall, the content of aroma was higher in Group C, while it was lower in Groups A and B. This indicates that the PEHC can delay the increase of aroma in fruits.

Thirty-three fatty acids (**Figure 14D**) were identified The overall performance of Group C was slightly lower than that of Groups A and B, but the difference was not very significant.

38 substances related to pigments or phenolics (**Figure 14E**) were identified, with Group A having the lowest relative content of fruit pigments or phenolics, followed by Group B, and Group C the highest, indicating that the PEHC could delay the increase of coloring in the fruit, with results similar to those shown in **Figure 9B**.

Thirty-two soluble sugars (**Figure 14F**) were identified, with the lowest content in group A, the next highest in group B, and the highest in group C. This indicates that the hormone compound could delay the elevation of soluble saccharides in the fruit, with results similar to those shown in **Figure 9A**.

### 3.8 Analysis of ethylene synthesis metabolic pathways in postharvest kiwifruit under PEHC

**Figure 15:**
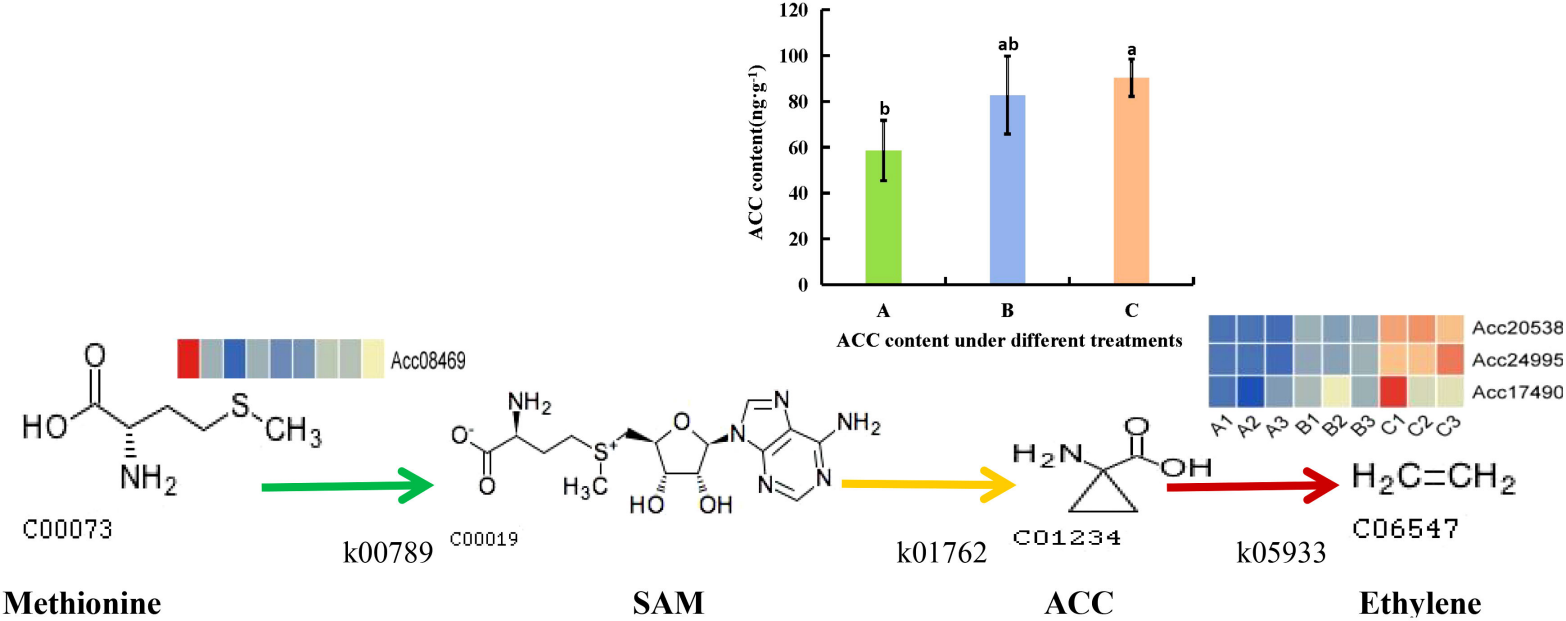
Changes in ethylene synthesis metabolic pathway after PEHC treatment. k00789, S-adenosylmethionine synthetase (metK). k01762, 1-aminocyclopropane-1-carboxylic (ACC) synthase(ACS). k05933, aminocyclopropanecarboxylate oxidase (ACO). SAM, S-adenosyl-L-methionine. ACC, 1-Aminocyclopropane-1-carboxylate. **(A)** kiwifruit stored at 4°C on the 2nd day. **(B)** kiwifruit stored at 4°C on the 30th day after PEHC treatment. **(C)** kiwifruit stored at 4°C on the 30th day. Different lowercase letters indicate significant differences between different treatments (p<0.05).

Kiwifruit belongs to the category of climacteric fruits, where the level of ethylene content directly affects the storage duration of the fruit. Therefore, we conducted an analysis on how PEHC influences the production of ethylene in kiwifruit. The PEHC inhibits ethylene production by down-regulating gene expression in ethylene metabolism, which in turn delays fruit softening (**Figure 15**). Particularly, the PEHC reduced the content of ethylene synthesis precursor (1-Aminocyclopropane-1-carboxylate, ACC) in kiwifruit at day 30 of storage and was 8.34% lower than the control. The PEHC treatment down-regulated *Acc08469* in S-adenosylmethionine synthetase (metK) and gene expression of *Acc20538*, *Acc24995*, and *Acc17490* in 1-aminocyclopropane-1-carboxylate oxidase (ACO).

### 3.9 Validation of RNA-Seq results by qRT-PCR

**Figure 16:**
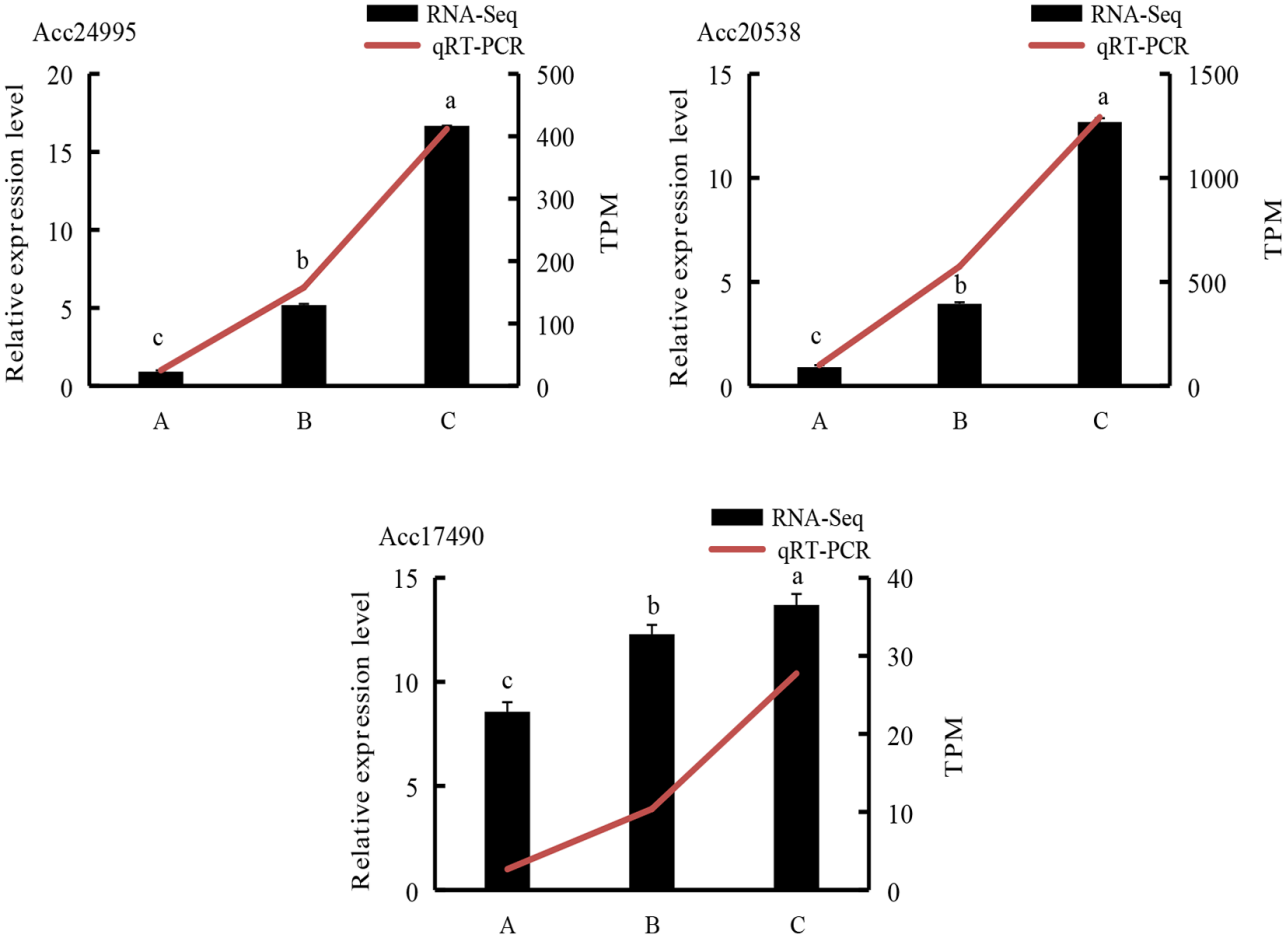
qRT-PCR analysis of representative genes. A, kiwifruit stored at 4°C on the 2nd day. B, kiwifruit stored at 4°C on the 30th day after PEHC treatment. C, kiwifruit stored at 4°C on the 30th day. Different lowercase letters indicate significant differences between different treatments (p<0.05).

To further validate the RNA-Seq results (**Figure 16**) and evaluate candidate genes involved in the ethylene biosynthesis pathway, we selected 3 DEGs. The qRT-PCR data for these genes were consistent with the RNA-Seq results, confirming the reliability of the transcriptome sequencing data.

## 4 Discussion

Fruit hardness, weight loss rate, and good fruit rate reflect the effect of fruit preservation, which is the main index of fruit quality (Geng et al., 2021). SSC and TAC are important indicators of kiwifruit fruit intrinsic quality (Tang, 2023). Under appropriate BR concentration treatment, the decrease in fruit hardness, good fruit rate, and SSC can be delayed, thereby reducing decay. Blueberries also showed the same results under BR treatment (Min et al., 2022). Su (2021) found that a certain concentration of MT can effectively inhibit the postharvest fruit respiration rate, weight loss rate, hardness, and color decline, and reduce kiwifruit rot and softening. MT at an appropriate concentration (200 μmol·L^-1^) can reduce the weight loss rate of kiwifruit and decrease the good fruit rate, hardness, and SSC (**Figure 2**). SA was a plant hormone that can delay ripening by regulating ethylene production or inhibiting ethylene synthesis. It can also reduce fruit decay and weight loss during storage. Using MeJA in post-harvest kiwifruit was beneficial for reducing weight loss, and maintaining good fruit rate, hardness, and SSC. This result was consistent with the research findings of predecessors on Chinese chestnuts (Uzun, 2023) and citrus fruits (Baswal et al., 2020). Previous studies have found that postharvest application of a certain concentration of SA can maintain the firmness and the rate of good fruit of blackberries (Sakaldaş et al., 2024), sweet cherry fruit (Yao and Tian, 2005), pears (Ali et al., 2022; Zhang et al., 2023), and apricots (Ezzat et al., 2020), thereby delaying fruit senescence. This may be related to the fact that SA induces the defensive resistance system of fruits during the storage process. This might have been related to the fact that SA induced the defensive resistance system of fruits during the storage process. Our study showed that SA was beneficial for maintaining a higher rate of good fruit, hardness, and SSC, while reducing the weight loss rate (**Figure 4**). This was consistent with the results of Baswal et al (Baswal et al., 2020), who found that treatment with SA (0.002 μmol·L^-1^) could maintain a higher hardness and SSC, as well as reduce weight loss in citrus fruits. The optimal hormone concentration treatment was effective in the storage and preservation of kiwifruit, not only in increasing the percentage of good fruit rate, and hardness but also in reducing the weight loss rate and disease index. In this study, lower weight loss rate, delayed accumulation of SSC, and higher good fruit rate and hardness were found at BRs, MT, MeJA and SA concentrations ranging from 5 to 15 μmol·L^-1^ (**Figures 1A-D**), 200 μmol·L^-1^ (**Figures 2A-D**), 300 μmol·L^-1^ (**Figures 3A-D**) and 2 mmol·L^-1^ (**Figures 4A-D**), respectively.

Some studies found that kiwifruit with the extension of storage time pulp brightness gradually reduced, the color from green to light yellow, the use of BRs can slow down the decline in brightness and chlorophyll decomposition, and maintain the fresh color of the fruit (Wang, 2020). The kiwifruit TAC of PEHC was slow to decrease compared to the control and was effective in maintaining the reduction of fruit VC content. Cai (2013) found that a certain concentration of SA had the best storage effect on postharvest kiwifruit ‘Feng Lv’, effectively reducing nutrient consumption and protecting the sensory quality of the fruit. Pan et al. (2019) showed that MeJA fumigation treatment of ‘Jinkui’ kiwifruit storage quality can effectively alleviate the decline of fruit SSC, TAC, and VC content, which is conducive to the maintenance of kiwifruit taste and characteristic flavor, and delay the loss of nutrients in the fruit. Lu (2018) treated ‘Huayou’ kiwifruit with 5 and 8 μmol·L^-1^ BRs, and found that 5 μmol·L^-1^ BRs effectively delayed the increase in weight loss rate and SSC of kiwifruit, and maintained fruit firmness and inhibited postharvest aging of fruits. MT not only acts as a signaling molecule involved in regulating the postharvest physiological metabolism of fruits and vegetables, but also maintains better nutritional quality of fruits and vegetables, improves total sugar content and energy level of fruits and vegetables after harvesting, and improves disease resistance of fruits and vegetables (Qu et al., 2022). The combined treatment of MeJA and SA enhanced the firmness, soluble solids content, and titratable acidity of postharvest apricot fruits (Ezzat et al., 2017). Kiwifruit fruit hardness, good fruit rate, TAC, and VC content decreased slowly after PEHC treatment (BRs concentration of 10 μmol·L^-1^, MT concentration of 200 μmol·L^-1^, MeJA concentration of 300 μmol·L^-1^, SA concentration of 2 mmol·L^-1^) compared with the control, and were able to effectively maintain higher fruit hardness and color, and delayed the increase of weight loss rate and disease index.

Based on metabolomics, a total of 1,552 differential metabolites were identified by qualitative and quantitative analysis of the effects of PEHC on kiwifruit metabolites during storage. Under 30 days of storage, PEHC increased the relative contents of acids and amino acids and decreased the relative contents of odors, pigments, and soluble sugars compared with the control group, of which the trends of changes in acids and soluble sugars were consistently associated with the changes in fruit quality. It showed that PEHC could improve the fruit storage quality, thus delaying the aging and softening of the fruit, and thus achieving the purpose of preservation. Some studies have shown that phytohormones can effectively inhibit ethylene biosynthesis in fruits, which is an important means of maintaining fruit quality and prolonging the storage period. For example, BRs (Hansen et al., 2009), MT (Zhai et al., 2018; Liu et al., 2019; Cheng et al., 2022), MeJA (Wu et al., 2020), and SA (Yin et al., 2013), and their mechanisms mainly focus on the inhibition of gene expression and enzyme activity. It has been demonstrated that hormone treatments inhibit the expression of genes involved in ethylene biosynthesis, such as *ACS1*, *PcACO*, *MaACO1*, and *ACO1*, and reduce ethylene production (Liu et al., 2019). The expression of the *Acc08469* gene in metK and *Acc20538*, *Acc24995*, and *Acc17490* genes in ACO was down-regulated by PEHC treatment as found by transcriptomics, and the ACC content was measured by targeted metabolism-phytohormone and was found to be lower in PEHC treatment as compared to control. ACS is a rate-limiting enzyme that has an important effect on ACC synthesis, and the decrease in ACC content in PEHC treatment in this study may be related to the down-regulation of *Acc20538*, *Acc24995*, and *Acc17490* gene expression. Moreover, during the conversion of methionine to SAM by metK, the down-regulation of *Acc08469* gene expression of metK may affect the synthesis of SAM, which in turn affects the synthesis of ACC. ACC synthase (ACS) and ACC oxidase (ACO) are the two main enzymes for ethylene synthesis (Hansen et al., 2009; Khan et al., 2024). In this paper, in conjunction with the results of PEHC treatment to obtain the storage quality of kiwifruit, we hypothesized that PEHC treatment could inhibit ethylene production in kiwifruit fruits and that the *Acc20538*, *Acc24995*, and *Acc17490* genes in ACO were likely to be the key genes for ethylene reduction.

## 5 Conclusion

In summary, PEHC can inhibit the expression levels of key genes in the ethylene synthesis pathway, indirectly reducing the ethylene content in kiwifruit, thus greatly improving the storage capacity and quality maintenance of kiwifruit. In particular, the PEHC down-regulated *Acc08469* gene expression in metK and *Acc20538*, *Acc24995*, and *Acc17490* gene expression in ACO and reduced ACC content. Notably, the PEHC reduced the weight loss rate and disease index of kiwifruit, increased hardness, good fruit rate, TAC and VC content, maintained fruit color, and delayed the accumulation of SSC. Furthermore, PEHC altered the relative levels of metabolites(DAMs) in kiwifruit, specifically increasing acids and amino acids while decreasing the relative levels of aroma, pigments or phenolics, and soluble sugars. Therefore, PEHC has great potential for the development as a green preservative for kiwifruit and even for other horticultural crops.

## Data Availability

The original contributions presented in the study are publicly available. This data can be found here: National Genomics Data Center, accession CRA025100. The original contributions presented in the study are included in the article/supplementary material, further inquiries can be directed to the corresponding author/s.
